# *Tryptophan hydroxylase* Is Required for Eye Melanogenesis in the Planarian *Schmidtea mediterranea*

**DOI:** 10.1371/journal.pone.0127074

**Published:** 2015-05-27

**Authors:** Bramwell G. Lambrus, Olivier Cochet-Escartin, Jiarong Gao, Phillip A. Newmark, Eva-Maria S. Collins, James J. Collins

**Affiliations:** 1 Howard Hughes Medical Institute and Department of Cell and Developmental Biology, University of Illinois at Urbana-Champaign, Urbana, Illinois, United States of America; 2 Neuroscience Program, University of Illinois at Urbana-Champaign, Urbana, Illinois, United States of America; 3 Division of Biological Sciences and Department of Physics, UC San Diego, San Diego, California, United States of America; University Zürich, SWITZERLAND

## Abstract

Melanins are ubiquitous and biologically important pigments, yet the molecular mechanisms that regulate their synthesis and biochemical composition are not fully understood. Here we present a study that supports a role for serotonin in melanin synthesis in the planarian *Schmidtea mediterranea*. We characterize the tryptophan hydroxylase (*tph*) gene, which encodes the rate-limiting enzyme in serotonin synthesis, and demonstrate by RNA interference that *tph* is essential for melanin production in the pigment cups of the planarian photoreceptors. We exploit this phenotype to investigate the biological function of pigment cups using a quantitative light-avoidance behavioral assay. Planarians lacking eye pigment remain phototactic, indicating that eye pigmentation is not essential for light avoidance in *S*. *mediterranea*, though it improves the efficiency of the photophobic response. Finally, we show that the eye pigmentation defect observed in *tph* knockdown animals can be rescued by injection of either the product of TPH, 5-hydroxytryptophan (5-HTP), or serotonin. Together, these results highlight a role for serotonin in melanogenesis, perhaps as a regulatory signal or as a pigment substrate. To our knowledge, this is the first example of this relationship to be reported outside of mammalian systems.

## Introduction

Melanin is a biologically important pigment found throughout the animal kingdom. The two major, chemically distinct groups of melanins are the brown-to-black eumelanins and the yellow-to-reddish pheomelanins [1–3], and it is the quantity and quality of these pigments that determine the color of the hair, skin, and eyes of animals [4–6]. Various organisms employ these pigments in diverse and vital roles, such as camouflage, sexual attraction, and photoprotection [1–3,7–9]. Despite their significance, melanins remain poorly characterized due to their insolubility and their extreme molecular heterogeneity [3–6]. Furthermore, the regulatory mechanisms that control their biosynthesis remain poorly understood [10]. To better understand the diverse constituency of melanins, it is important to study their composition and the regulation of their biosynthesis in model organisms.

One model that may inform us about melanin synthesis is the planarian *Schmidtea mediterranea*. Planarians are free-living flatworms that are renowned for their regenerative abilities [11]. There are two distinct types of pigmentation in the planarian body: a black pigment of the eyes, and a lighter brown pigmentation throughout the surface of the body [12]. Previous histological and biochemical studies have shown that the black eye pigment in the planarian pigment cup is composed of eumelanin, while the body pigment is composed of non-melanin pigments [12,13]. The work in this study centers on the black pigment of the planarian eye. The planarian visual system consists of only two cells types: 1) the light-sensitive photoreceptors, and 2) a layer of pigment-producing cells which envelops the light-sensitive region of the photoreceptors, termed the pigment cup.

*S*. *mediterranea* has in recent years emerged as a tractable model for studying regeneration and other processes, owing to a sequenced genome and the robust function of molecular tools such as whole-mount *in situ* hybridization and RNA interference (RNAi) [11]. During a regeneration screen of neural genes in *S*. *mediterranea*, we tested the functional role of *tryptophan hydroxylase* (*tph*), which catalyzes the rate-limiting step of serotonin synthesis and has previously been identified in the planarian nervous system [14]. Tryptophan hydroxylase adds a hydroxyl group to the 5 position of tryptophan to form 5-hydroxytryptophan (5-HTP), which is subsequently decarboxylated to form 5-hydroxytryptamine (5-HT, or serotonin). After disrupting *tph* function in *S*. *mediterranea* by RNAi, we observed an unexpected phenotype: the planarians regenerate eyes that lack signs of a pigment cup.

Disrupting the serotonin synthesis pathway has not been previously reported to result in impaired eye development or pigment synthesis. Here, we investigate this putative role of serotonin by characterizing the phenotype resulting from *tph* knockdown in *S*. *mediterranea*. We employ immunohistochemistry and electron microscopy to assess the results of TPH deficit at a tissue and ultrastructural level. We also exploit the *tph*(*RNAi)* phenotype to test the biological function of the pigment cup in planarians. This primitive structure has long been presumed to provide directionality to the worms by shielding their photoreceptors, but this hypothesis has remained untested [15]. We performed behavioral analysis using a photophobic assay and show that, despite lacking eye pigment, *tph* knockdowns remain able to orient away from a light source, though at a significantly slower rate relative to controls. This suggests that while eye pigment facilitates phototaxis, these planarians have more than one mechanism of light detection. Finally, we successfully rescue the eye pigment by injecting *tph* knockdown animals with the downstream products of TPH activity. Together, our results point to a novel role for serotonin in melanin synthesis in the planarian *Schmidtea mediterranea*.

## Materials and Methods

### Planarian Culture

Clonal lines of asexual *S*. *mediterranea* were used for all experiments and were maintained at 20°C in 1.0x Montjuïc salts [16]. Animals were fed weekly with organic calf liver paste. To minimize non-specific gut background from feeding, animals were starved for at least 1 wk prior to fixation for *in situ* hybridization and immunohistochemistry.

### Chemicals

Reagents were obtained from Sigma-Aldrich (St. Louis, MO) unless stated otherwise.

### Gene Sequence

**Fig 1:**
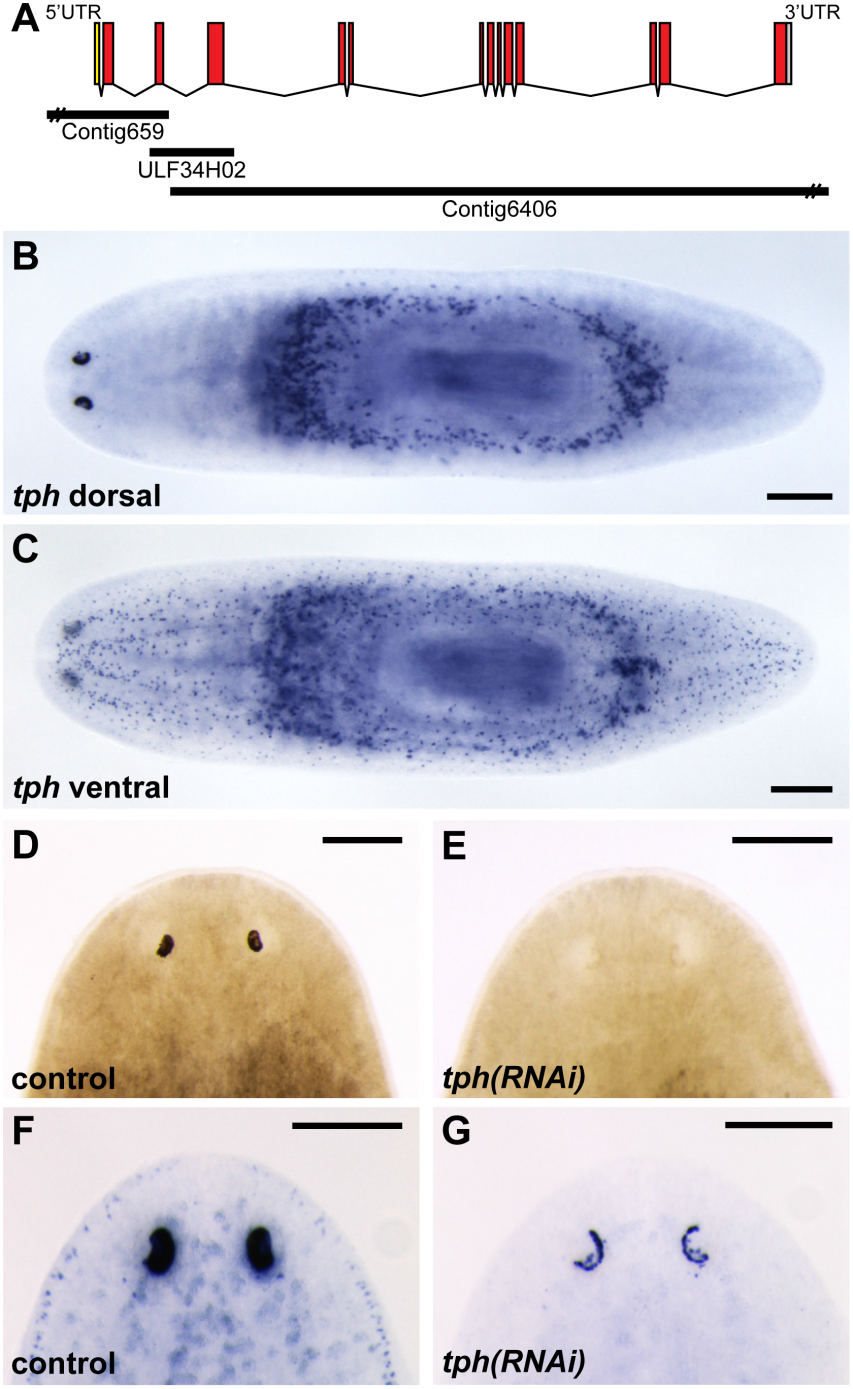
*tph* is essential for photoreceptor pigmentation after regeneration. (A) Structure and genomic organization of the *tph* gene. Top, gene structure of *tph*; the 5’-untranslated region, the coding region, and the 3’-untranslated region are depicted in yellow, red and blue, respectively. Below, organization of genomic regions (supercontigs) that encode *tph*. (B-C) Whole-mount *in situ* hybridization to localize *tph* expression. The *tph* gene was expressed in the pigment cups, the peripharyngeal secretory cells (dorsal) and cells within the central and peripheral nervous systems (ventral). (D-E) RNAi-mediated knockdown of *tph*. In comparison to controls (D), *tph* knockdowns (E) regenerate pigment cups that appear to lack pigment; 21-day regenerates shown. (F-G) *in situ* hybridizations to detect *tph* mRNA levels following RNAi treatment. Relative to controls (F), *tph* dsRNA-treated animals show dramatically reduced *tph* mRNA expression; 21-day regenerates shown. Scale bars 200 μm.

To determine the full-length sequence of the *S*. *mediterranea tph* gene, EST [17] and 5’ Rapid Amplification of cDNA Ends (RLM-Race, Ambion, Austin, TX) sequences were assembled using Sequencher 4.7 (Gene Codes, Ann Arbor, MI). These sequence data were aligned to the *S*. *mediterranea* genome to determine the structure of the *tph* gene. While the 5’ and 3’ ends of *tph* aligned to supercontigs 654 and 6406, respectively, we found 30 nucleotides in exon three that showed no similarity to any sequences in the current *S*. *mediterranea* genome assembly. Thus, we used this 30 nucleotide sequence to query S. *mediterranea* genome sequence reads present in the NCBI trace archive. This analysis identified a single trace (ULF34H07) that included the missing sequence data. Assembly of this trace with supercontigs 654 and 6406 collapsed these sequences into a single contig (Fig 1A), which represents the only *tph* gene in this species.

### RNAi

For some RNAi experiments, animals were fed bacterially expressed dsRNA, essentially as described in [18]. Templates were first cloned into pJC53.2 [19], then transformed into RNaseIII-deficient bacterial strain HT115(DE3) cells [20]. 6 mL of IPTG-induced culture were pelleted, frozen at -80°C, and resuspended in 150 μL calf liver paste. To generate knockdowns, animals were fed this dsRNA mixture 3 times over nine days, cut 3 days after the last feeding, and allowed to regenerate for 10 days. A non-homologous *C*. *elegans unc-22* gene was used as the control. To image the phenotype, regenerates were killed in 5% N-acetylcysteine (NAC), fixed in 4% formaldehyde, mounted in glycerol and imaged using a Leica DFC420 camera mounted on a Leica M205A stereomicroscope (Leica, Wetzlar, Germany). In other experiments, knockdowns were generated by feeding *in vitro* transcribed dsRNA, as previously described [19], and using the *ccdB* and *camR*-containing insert of pJC53.2 as a control. 4–8 μg of dsRNA were combined with 40 μL of a 3:1 liver:water mixture, and worms were fed and amputated following the same timeline as described above. Identical results were obtained whether animals were fed bacterially expressed or in vitro-synthesized dsRNA.

### In Situ Hybridization and Immunohistochemistry

Whole-mount in situ hybridization was performed with a formaldehyde-based fixation procedure as previously described [21]. Samples were imaged with a Leica DFC420 camera mounted on a Leica M205A stereomicroscope (Leica, Wetzlar, Germany), equipped with a Leica TL RC base. For fluorescent *in situ* hybridization (FISH), following post-hybridization washes and blocking, animals were incubated in α-Digoxigenin-POD (1:1000, Roche, Mannheim, Germany) overnight at 4°C. Samples were then washed in MABT for 2 h, equilibrated in TNT (100 mM Tris pH 7.5, 150 mM NaCl, and 0.05% Tween-20), and developed with Cy3-tyramide (TSA-Plus, Perkin Elmer, Waltham, MA) following the manufacturer’s protocol. Samples were treated with 4’,6-diamidino-2-phenylindole (DAPI) to stain nuclei, mounted in Vectashield (Vector Laboratories, Burlingame, CA) and imaged on a Zeiss LSM 710 confocal microscope (Carl Zeiss, Germany). DAPI and Cy3 were excited with 405 nm and 561 nm lasers, respectively. Images were processed using either Zen 2008 (Carl Zeiss, Germany) or ImageJ [22]. To visualize planarian photoreceptors, FISH samples were further incubated in immunohistochemistry blocking buffer (0.6% BSA and 0.45% fish gelatin in PBS and 0.3% Triton X-100) for 1 h, incubated overnight in monoclonal antibody VC-1 that labels the visual neurons [23] (diluted 1/16000) at 4°C, washed in PBSTx, and incubated in Alexa Fluor 488-conjugated goat-anti-mouse IgG (Invitrogen, Carlsbad, CA). The gene termed “*tyrosinase*” used in this study is similar to both *tyrosinase* and *dopachrome tautomerase* of higher organisms. The GenBank accession number for the *tyrosinase* sequence used in this study is AY067481.1.

### Electron Microscopy

To prepare samples for transmission electron microscopy (TEM), worms were treated as described in [24]. Whole animals were fixed with 2% formaldehyde and 2.5% glutaraldehyde in 70 mM Na-Cacodylate Buffer, 1 mM CaCl_2_, pH 7.4, for 10 min on ice. Animals were cut to isolate tissues of interest, and pieces were fixed for 4 h on ice. Samples were postfixed in 1% aqueous osmium tetroxide in the same buffer, in darkness, for 90 min at room temperature, then dehydrated in an ethanol series and embedded in Epon-Araldite. Cured blocks were sectioned using a Reichert Ultracut S Ultramicrotome; 80 nm sections were collected in 200-mesh hexagonal copper grids, stained with Venable's lead citrate [25] and imaged using a Phillips CM200 Transmission Electron Microscope at 120 kV. For pigment cup identification, thick survey sections (1 μm) were collected on glass slides, stained with 1% Toluidine Blue in 2% Borax and observed with a light microscope.

### Planarian Tracking

To analyze photophobic ability, planarians were individually placed in a tracking arena and their locomotion was recorded in response to a light gradient, similar to previous studies [23]. The photophobic response of control and *tph(RNAi)* worms was assessed under 3 different gradient intensities (low, medium, and high) as characterized in S1 Table, and the number of worms tested in each condition is listed in S2 Table. The tracking arena contained three black sides, to reduce reflections, with the last side being transparent to accommodate a white light LED panel (Amazon) used to generate the light gradient. The arena measured 14.5 cm in length, 6.2 cm in width, and 2.5 cm in depth, and was filled with 45 mL planarian water, which was exchanged between each set of experiments. Planarians were placed 5.5 cm from the front (transparent side) of the arena, and manually oriented to face the light source. To enable tracking, a cold cathode homogenous backlight panel (ML-0405, Edmund Optics) was placed underneath the arena at a distance of 21 cm, providing low-level general illumination for imaging. All light sources used in this assay are cool-running to minimize the possibility of the planarians sensing the light by the absorption of photons through their body pigment, which can dissipate the energy as heat.

A hand-held optical power meter (Model 1918-c, Newport) was used to determine the spatial light intensity. The homogenous light source was measured to be (9.7 ± 0.5) μW without shading (used in high and medium light gradients) and (6.8 ± 0.5) μW with shading (used in low light gradient conditions).

Images were acquired at 10 frames per second using a Basler A601f camera, equipped with a 25 mm lens (Edmund Optics), and using custom MATLAB scripts [26]. Image analysis was also performed in MATLAB using standard methods such as background removal and thresholding. For each run, center-of-mass tracking algorithms were applied until either the planarian left the field of view, or up to 90 s (900 frames) for the medium and low gradient setups and 50 s (500 frames) for the high gradient setup. From the center-of-mass tracking, we computed the orientation of the velocity θ(i,t) of the i^th^ worms at time t with respect to the direction of the gradient (θ = 0 indicating orientation towards high light intensity and θ = ± π towards low light intensity). Angular distributions of θ were then manually chosen for representative time periods for the different orientations (towards light, away from light, and random) for the two populations (S2 Table). For quantification, we defined an orientational order parameter for a given population as:

$$\text{S}\left(\text{t}\right)=<\text{cos}\left(\theta \left(\text{i,t}\right)\right){>}_{\text{I = 1:10}}$$

giving S(t) = +1 for perfect alignment towards the regions of high light intensity, S(t) = -1 for alignment in the opposite direction, and S(t) = 0 for random orientation. Standard errors of the mean (SEM) were calculated for the whole population of n = 10 animals.

### Microinjections

For rescue experiments, solutions of 100 mM tryptophan (Sigma, T0254), 100 mM 5-hydroxytryptophan (5-HTP) (Sigma, H9772), 2.5 M 5-HTP, 250 mM serotonin (Sigma, H9523), and 10 mM L-3,4-dihydroxyphenylalanine (L-DOPA) (Alfa Aesar, Ward Hill, MA) were prepared in vehicle (3:1 DMSO:MilliQ water). Control and *tph(RNAi)* worms were injected with 50 nL of the respective solutions or vehicle using a Drummond Nanoject II microinjector (Broomall, PA). The worms were imaged before injections, and indicated intervals after injections to monitor phenotype rescue, using a Leica MZ16FA stereo microscope (Leica Microsystems, Wetzlar, Germany) and Basler 601f CCD camera (Basler AG, Ahrensburg, Germany).

## Results and Discussion

### *tph(RNAi)* planarians regenerate heads that lack eye pigment

To characterize the *tph* gene in *S*. *mediterranea*, *tph* gene structure and mRNA sequence were determined (as described in Methods), and gene expression was characterized by whole-mount *in situ* hybridization (Fig 1A–1C). Similar to previous reports [14,27], *tph* expression was observed in the cephalic ganglia and cells associated with the ventral nerve cords, consistent with its role in producing the neurotransmitter serotonin. Furthermore, *tph* transcript was also present in the peripharyngeal secretory cells and the pigment cup of the eye (Fig 1B), where there are no clear roles for serotonin signaling.

To determine whether *tph* plays any functional role in the development or maintenance of the pigment cups, we performed RNA interference (RNAi) to disrupt *tph* function. Following dsRNA treatment, animals were decapitated approximately 400 μm behind the photoreceptors and allowed to regenerate. By 21 d after cutting, control animals developed eyes of normal appearance, while *tph(RNAi)* animals regenerated eyes without observable pigment cups (Fig 1D and 1E). Body pigmentation of *tph*(RNAi) worms was unaffected and appeared identical to that of control worms. Knockdown efficiency was assessed by comparing *tph* expression levels in controls and *tph* knockdowns. In situ hybridization analysis showed that *tph* mRNA levels were substantially reduced in *tph* knockdown animals, indicating that our RNAi treatment robustly inhibited *tph* expression (Fig 1F and 1G).

### Eye pigment is absent but cellular structures are preserved in *tph* knockdowns

**Fig 2:**
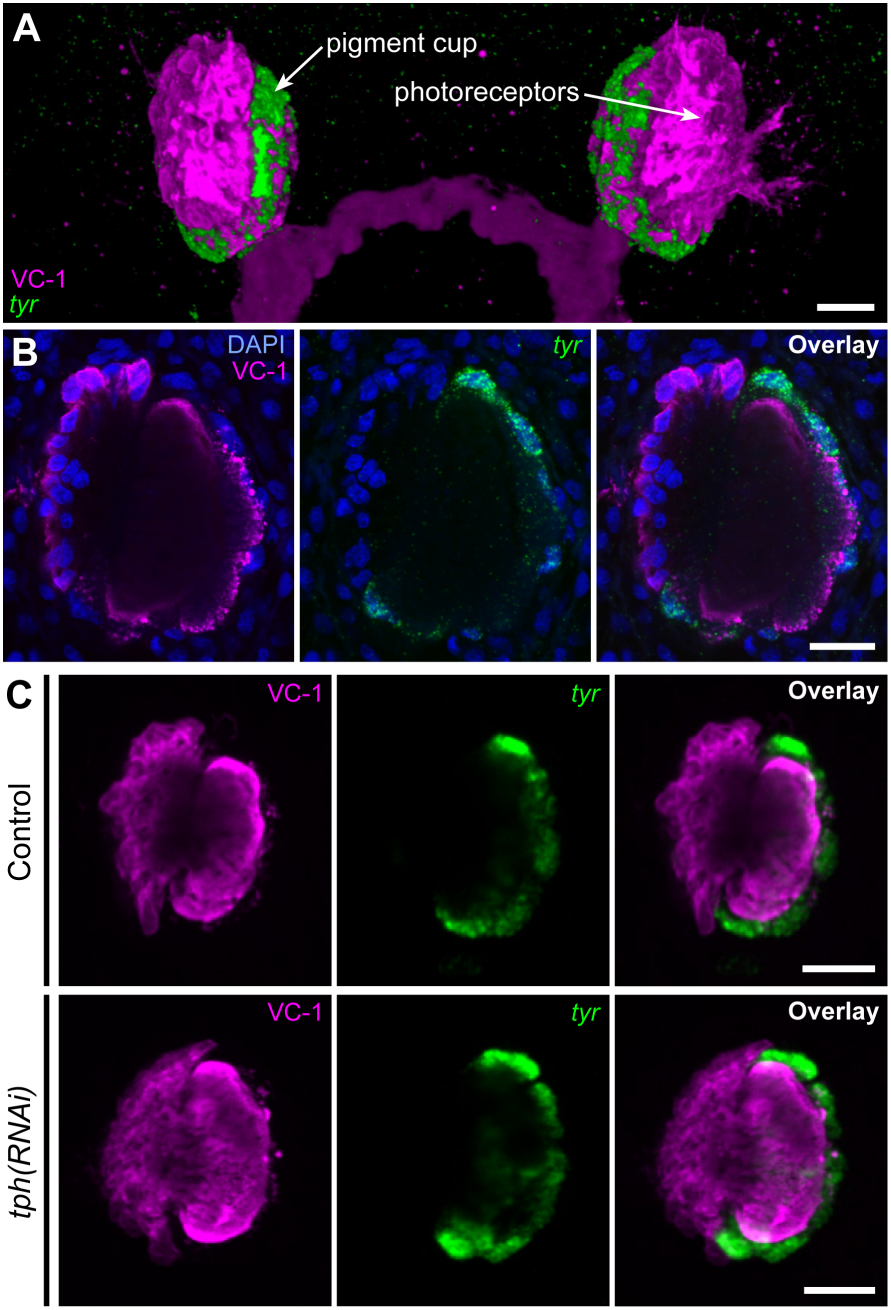
Pigment cup and photoreceptors remain intact in *tph(RNAi)* animals. (A-B) The planarian visual system. Photoreceptors (magenta) are visualized by immunofluorescence with VC-1 antibody against arrestin, and pigment cup cells (green) are visualized by fluorescent *in situ* hybridization of *tyrosinase (tyr)*. (A) depicts a maximum intensity confocal projection, whereas (B) represents a single confocal section. (C) By 10 days post-amputation, control and *tph(RNAi)* animals regenerate both photoreceptors and pigment cup cells, as indicated by VC-1 staining and *tyr* mRNA expression. Scale bars 20 μm.

To determine whether the *tph* phenotype is due to an absence of pigment cup tissue or a deficiency in pigment production, we labeled control and *tph* RNAi-treated animals with monoclonal antibody VC-1 against arrestin to detect photoreceptor cells [23], and with FISH against *tyrosinase (tyr)* mRNA, which is specifically expressed in pigment cup cells [28] (Fig 2A and 2B). Fluorescence microscopy revealed the presence of fully formed photoreceptor and pigment cup cells in both control and *tph* RNAi-treated samples 10 days post-amputation (Fig 2C). This shows that the lack of observed pigment cup structures in *tph* knockdowns is not due to a failure to regenerate pigment cup tissue, but due to a defect in pigmentation.

**Fig 3:**
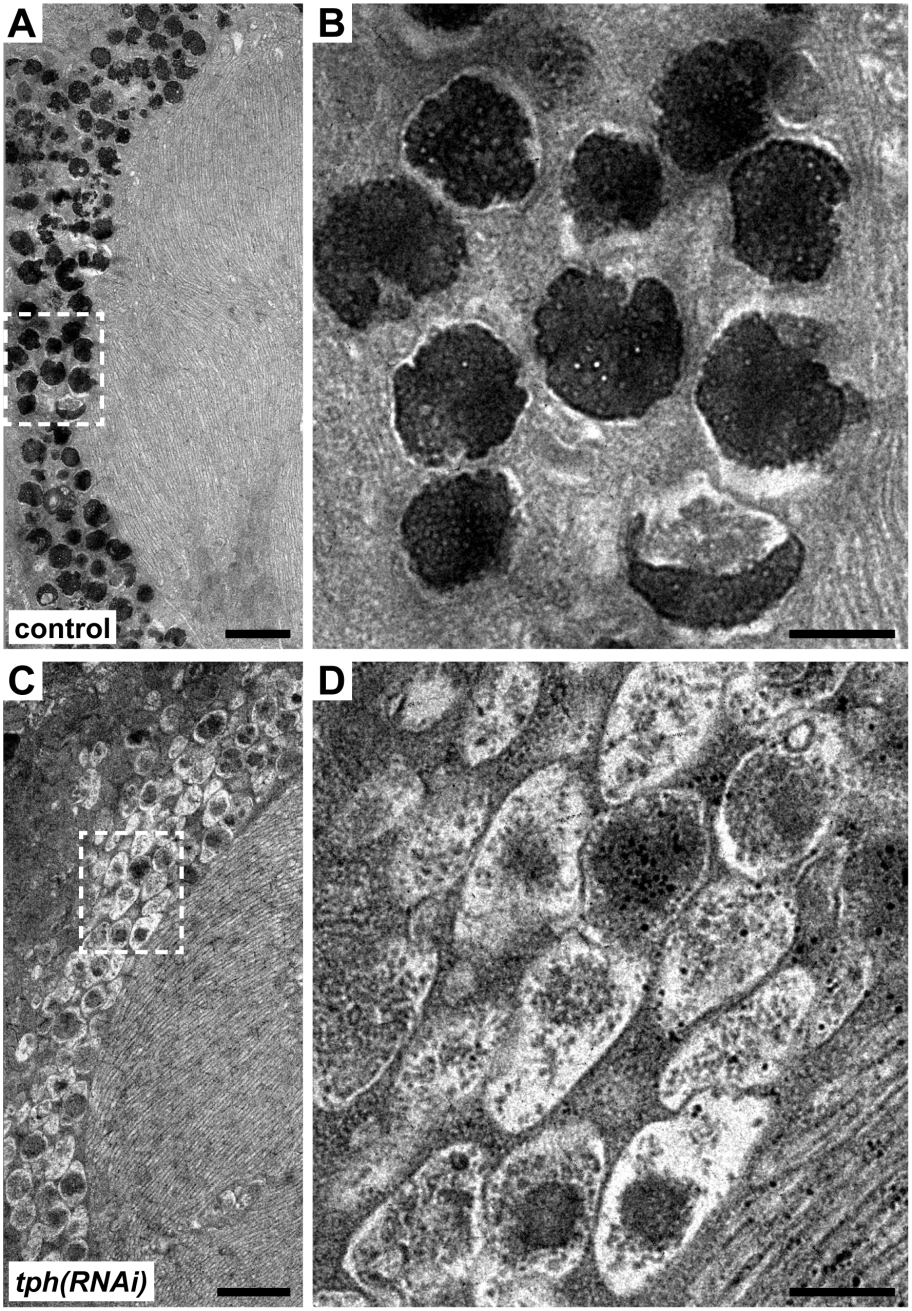
*tph(RNAi)* animals regenerate pigment cup cells largely devoid of mature melanosomes. All micrographs are transverse sections of the planarian eye. (A) Electron micrograph of the photoreceptor rhabdomes and the pigment cup cells in control RNAi animals. Panel (B) is a magnified view of the pigment cup cells highlighting the mature melanosomes. (C-D) Electron micrograph of the photoreceptors of *tph* knockdown animals. In *tph* knockdowns the photoreceptors and the pigment cups are intact, but the melanosomes appear immature and less electron dense compared to controls. Scale bars in A and C are 2000 nm; in B and D are 500 nm.

To characterize the nature of this pigment deficiency, RNAi-treated samples were fixed and sectioned for electron microscopy. The planarian photoreceptors are distinguishable by a highly ordered microvilli-containing structure called the rhabdome (Fig 3A), which is responsible for receiving and transducing light stimuli. The rhabdome is encompassed by a single layer of pigment cup cells which contain numerous spherical, electron-dense pigment granules housed in organelles termed melanosomes. These melanosomes are distributed in the cytoplasm, toward the interior surface of the cup (Fig 3A) [29]. In control RNAi animals, we observed melanosomes of the expected round shape and high electron density (Fig 3A and 3B). In contrast, micrographs of *tph(RNAi)* animals revealed a strikingly different phenotype. Specifically, melanosomes in *tph* knockdowns were dramatically reduced in electron density (Fig 3C and 3D); many contained only faint specks of electron-dense material and displayed less overall electron-density than the surrounding cytoplasm. Furthermore, unlike the spherical melanosomes observed in controls, the melanosomes of *tph* RNAi-treated animals appeared flattened and more closely packed. The results of these ultrastructural studies indicate that the phenotype of *tph* RNAi-treated animals is a result of a deficiency in pigment synthesis, rather than in the development of cellular or subcellular structures.

### *tph* knockdown decreases the efficiency of the planarian photophobic response

The pigment cup is a primitive eye structure that, in addition to serving a photoprotective role, is believed to enable an organism to detect the direction of incident light and thus achieve phototaxis [15]. To our knowledge, however, this hypothesis has not yet been directly tested. We sought to exploit the *tph* phenotype as an example of a functionally deficient pigment cup. If the hypothesized role of the pigment cup is accurate, we would expect to see a loss of phototaxis ability in *tph* knockdowns due to their unpigmented cups. Planarians are distinctly photophobic and robustly move away from light. Taking advantage of this reproducible behavior, we developed a light-avoidance assay to quantitate behavioral changes in *tph* knockdowns.

**Fig 4:**
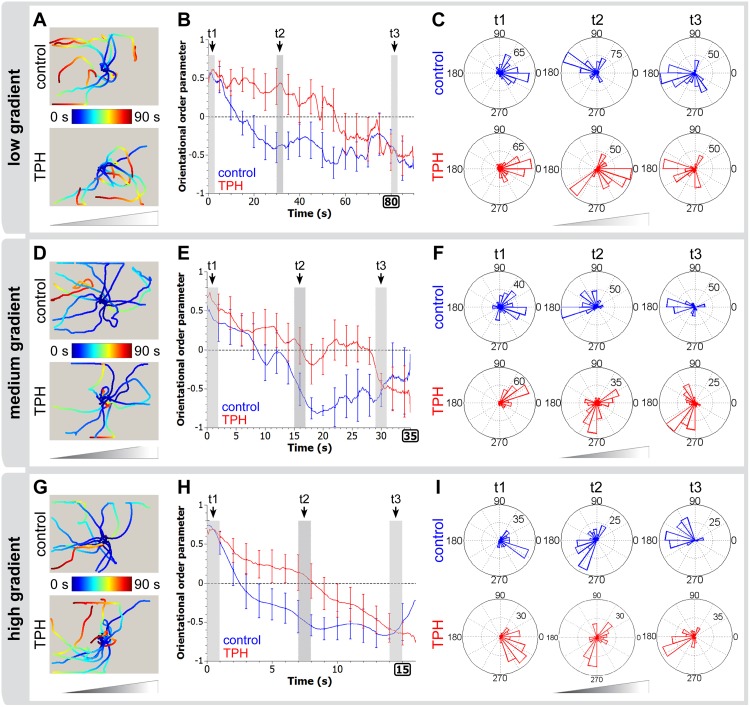
*tph(RNAi)* animals lacking eye pigment are slower to orient to light. Orientation dynamics of control and *tph(RNAi*) planarians under low (A-C), medium (D-F) and high (G-I) light gradient conditions. Both control and *tph(RNAi)* worms respond to all three gradients by turning away from the light source, but *tph(RNAi)* worms react less efficiently, especially in low light gradients. (A, D, G) Center-of-mass tracking results for both control and tph(RNAi) populations (n = 10). Triangles indicate the orientation of the light gradient, with the light source on the right. All points are color-coded for time, as shown by color bar legends. At t = 0 s, the worms are manually oriented toward the light source. (B, E, H) Orientational order parameters as a function of time for both populations (see Methods). The error bars show SEM at selected time points. The dashed y = 0 line is a guide for the eye showing the threshold between orientation biased toward the light souce (y > 0) and away from the light source (y < 0). Note the decrease in time scale as the gradient strength is increased, indicating a faster negative phototactic response of both populations at increased illumination. (C, F, I) Angular distributions of velocities at different time periods (corresponding to grayed regions in B, E and H) showing the reversal of polarity at different rates between control and *tph(RNAi*) worms. The size of the wedge indicates the proportion of worms traveling in the given direction in that time period, where 0° is towards the light source, and 180° is away from the light source. A two-way ANOVA test confirms statistical difference at the 1% level for TPH and control animals as well as for the three different gradient settings.

The photophobic responses of control and *tph(RNAi*) worms were tested under light gradients of different intensities (low, medium, and high), as described in Methods. Each planarian was individually tracked in an arena, to avoid possible interactions or collective behaviors. At the start of the assay (t = 0 s), each planarian was manually oriented towards the light source to minimize possible dependence of their response upon the original orientation and to promote reproducibility. The light source was then switched on, and their behavior recorded as the planarian moved freely in the arena (Fig 4A, 4D and 4G).

For all three gradient settings, we found that both control and *tph(RNAi*) worms were able to orient away from the light source on the order of tens of seconds (Fig 4B, 4E and 4H). This behavior is quantitatively described by the increasingly negative values of the orientational order parameter S(t), which uses the following metric: a value of “1” indicates traveling directly towards the light source, and a value of “-1” indicates locomotion in 180 degrees the opposite direction (see Methods). We also found that the higher the intensity of the light gradient, the faster the planarians were able to establish negative orientation. In the absence of a gradient, neither *tph* nor control RNAi worms exhibited directional biases (data not shown). These results show that both sample groups were able to perceive and respond to the presence of the light gradient, and suggest that the shielding function of the pigment cup is not essential for directional perception in these worms.

However, while *tph(RNAi*) worms retained a photophobic response, close inspection revealed that there were significant differences in the rate of re-orientation in control and *tph(RNAi*) populations. Compared with control worms, *tph* knockdowns exhibited a slower re-orientation in response to the gradient (Fig 4B and 4C, 4E and 4F, 4H and 4I). This effect was apparent in all three gradient settings. To quantify this effect, we defined an orientation time τ as the time necessary for the population to reach an orientational order parameter of -0.5 or less, indicative of a strong orientation away from the light source. For control RNAi and *tph(RNAi)* worms, we measured, respectively, τ = (500020030± 20) s and τ = (76.7 ± 16) s for the weak light gradient setup, τ = (16 ± 2) s and τ = (31.9 ± 3) s for the medium light gradient and τ = (7.7 ± 2) s and τ = (13.5 ± 3) s for the strongest gradient, showing that *tph* knockdown animals require 1.5- to 2-fold more time to re-orient to light relative to controls. To ensure that this effect is due to the lack of eye pigment in *tph*-knockdowns and not due to other effects of *tph* RNAi, we analyzed head pieces of *tph(RNAi*) worms, which retain their original eye pigment. These worms were found to behave like controls, confirming that the previously observed behavioral phenotype was due to the effect of *tph(RNAi)* on the pigment cup and not to other effects from serotonin deficit (S1 Fig). Together, these results show that while eye pigment is not essential for sensing light directionality and phototaxis in *S*. *mediterranea*, it does promote the efficiency of this response.

One explanation for the observed dynamics is that the planarian evolved to depend on multiple mechanisms for the detection of light, of which the pigment cup plays one role. An alternative mechanism is illustrated by the observation that certain species of planarians retain their photophobic response even after amputation [30,31], supporting the existence of somatic light-sensitive receptors. Further studies would be required to investigate the role of such compensating processes in the photophobic response, towards a better understanding of light-sensing mechanisms.

Interestingly, previous studies have reported that serotonin is required for ciliary locomotion in planarians [27]. We did not observe defects in ciliary locomotion in *tph(RNAi)* worms (S1 Fig). This is likely due to the incomplete nature of RNAi, which does not deplete serotonin production below the threshold required for ciliary function. In summary, we were able to parse out the contribution of the pigment cup to phototaxis by using the *tph(RNAi*) phenotype.

### *tph* RNAi eye phenotype is rescued by 5-HTP and serotonin injection

**Fig 5:**
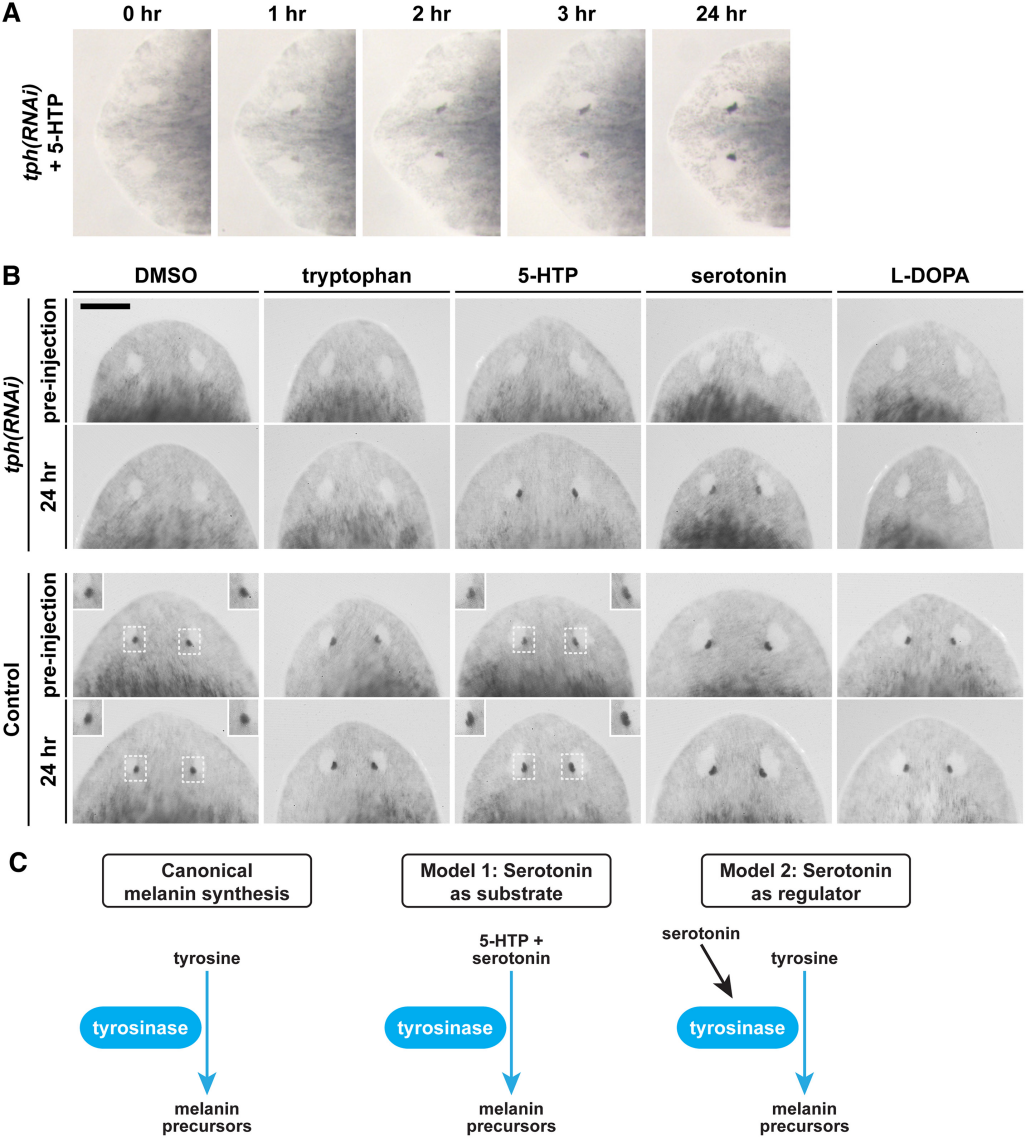
Tryptophan derivatives rescue eye pigment in *tph* knockdowns. (A) Progress of pigment recovery after injection of 2.5 M 5-HTP is shown at hour-intervals. Eye pigment in *tph* knockdowns is largely recovered by 3 hr post-injection. (B) *tph(RNAi*) and control worms were injected with DMSO (n = 3), 100 mM tryptophan (n = 3), 100 mM 5-HTP (n = 5), 250 mM serotonin (n = 5), or 10 mM L-DOPA (n = 3). Images show phenotype at pre-injection and 24 hr time points. Insets highlight the increased pigmentation of control worms injected with 5-HTP vs. DMSO control. Scale bar is 200 μm. (C) Comparison of traditional and hypothesized melanin synthesis pathways.

We next characterized the biochemical basis of the phenotype. To test if the direct product of the *tph* gene, L-5-hydroxytryptophan (5-HTP), is required for pigment synthesis in the planarian pigment cup, we microinjected 7-day *tph(RNAi*) regenerates with 5-HTP and assessed whether eye pigment would be rescued. By 1 hr post-injection, a discernable amount of eye pigment began to develop in *tph* knockdowns (Fig 5A), and at 3 hrs post-injection, eye pigment was nearly completely restored. By 24 hrs post-injection, *tph* knockdowns were identical in appearance to the control worms. Control worms that were injected with 5-HTP also appeared to experience a slight increased darkening of eye pigments (Fig 5B). *tph(RNAi*) and control worms that were injected with carrier DMSO alone did not experience darkening of eye pigment (Fig 5B).

To gain further insight into the nature of the *tph* phenotype, we also tested whether the relevant compounds 5-hydroxytryptamine (serotonin) or L-3,4-hydroxyphenylalanine (L-DOPA), a substrate for canonical melanin synthesis [10], could rescue eye pigment. Injections of 250 mM of serotonin were also able to rescue the *tph* phenotype (Fig 5B), and led to rapid return of pigment. In contrast, injections of 10 mM L-DOPA did not result in eye pigment recovery in *tph* knockdowns. We also tested whether injecting the knockdowns with 100 mM tryptophan could overcome the deficiency in TPH, but this treatment also did not result in eye pigment rescue. Combined, these results suggest that the components of the serotonin pathway—the precursor 5-HTP and serotonin itself—are essential for melanogenesis in the planarian *S*. *mediterranea*.

### Tryptophan hydroxylase function is required in *S*. *mediterranea* melanogenesis

Our results show that tryptophan hydroxylase is essential for the generation of eye pigment in *S*. *mediterranea*. Histological observations and biochemical analyses indicate that planarian eye pigment is composed of melanin [12,32]. Canonical melanin formation requires the activity of the enzyme tyrosinase, which catalyzes the oxidation of tyrosine to dihydroxyphenylalanine (DOPA) and then to dopaquinone, a precursor of melanin (Fig 5C) [2,33,34]. Indeed, *tyrosinase* is expressed specifically in the planarian pigment cup, consistent with previous indications of a melanin-based eye pigment [28]. Since the canonical melanin synthesis pathway utilizes tyrosine as a precursor, it is unexpected that disrupting an enzyme involved in tryptophan metabolism would inhibit melanin formation.

Interestingly, it has been shown that both the product of TPH, 5-hydroxytryptophan, and its derivative 5-hydroxytryptamine (serotonin) can form melanin when subjected to the action of tyrosinase *in vitro* [35], indicating that melanogenesis from tryptophan substrates is chemically possible. Because our data show that TPH is responsible for generating an essential component for pigment synthesis in *S*. *mediterranea*, one possible explanation is that the melanogenesis pathway of these worms relies exclusively on 5-hydroxytryptophan and its derivatives as substrates. Consistent with this scenario, it has been shown that tyrosinase is also required for eye pigment production in *S*. *mediterranea* [28]. Based on these observations, we propose an alternative model for melanogenesis in planarians, in which tyrosinase uses tryptophan derivatives (e.g. 5-hydroxytryptophan and/or 5-hydroxytryptamine) to generate melanin (Fig 5C). While previous studies have shown that a considerable degree of tryptophan can be incorporated into melanin, as in the case of mouse melanoma [35], it has not been shown that tryptophan can completely replace tyrosine as the precursor in melanogenesis. Therefore, this model would be unique in relying solely upon tryptophan derivatives for melanin synthesis.

An alternative explanation for this phenomenon is that, rather than serving as a substrate, serotonin signaling may be required to regulate melanogenesis in the pigment cup in *S*. *mediterranea*. Recent studies have shown that, in certain mammalian cell lines, serotonin signaling increases melanin synthesis through the action of serotonin receptors [36,37]. Regulation may also be mediated by melatonin, a downstream product of serotonin, which has also been reported to regulate pigment synthesis, although usually as an attenuator [34,38]. The mechanisms behind these forms of regulation remain poorly understood. Since little is known about either the conversion of tryptophan to melanin *in vivo*, or the mechanism by which serotonin or its downstream metabolic products activate melanogenesis, future studies should be aimed at dissecting the molecular basis for this phenomenon.

## Supporting Information

S1 Fig *tph(RNAi)* animals that retain original eye pigment do not demonstrate delayed phototaxis. Comparison of orientation dynamics of control (blue) and *tph(RNAi)* (red) head regenerates in high (A) and medium (B) gradient settings (see Methods). *tph(RNAi)* head regenerates possess pigment cups and show similar dynamics to control worms under the two gradient settings. In contrast, the *tph(RNAi)* tails, which lack pigment cups, exhibited slower re-orientation as shown in Fig 4 in the main text. Error bars show SEM. The line at y = 0 serves as a guide to show when the worms orient away from the gradient (y < 0).
(DOCX)

S1 Table Light intensities of gradients used in light-avoidance assay. Measurements were taken at the front end of the arena, at the planarian start location, and at the back end.
(DOCX)

S2 Table Number of trajectories used for the angular distributions at time points t1, t2, and t3 in Fig 4. A total of 10 worms per sample were run in each gradient condition. Due to boundary effects, some trajectories could not be included in the velocity analysis at the later time points.
(DOCX)
