# Supplementary material for: Tryptophan hydroxylase Is Required for Eye Melanogenesis in the Planarian Schmidtea mediterranea
Source: PLoS One. 2015 May 27;10(5):e0127074. doi: 10.1371/journal.pone.0127074 (PMC4446096; doi:10.1371/journal.pone.0127074)
Supplement: S1 Fig — Comparison of orientation dynamics of control (blue) and tph(RNAi) (red) head regenerates in high (A) and medium (B) gradient settings (see Methods). tph(RNAi) head regenerates possess pigment cups and show similar dynamics to control worms under the two gradient settings. In contrast, the tph(RNAi) tails, which lack pigment cups, exhibited slower re-orientation as shown in Fig 4 in the main text. Error bars show SEM. The line at y = 0 serves as a guide to show when the worms orient away from the gradient (y < 0). (DOCX) [file pone.0127074.s001.docx]

**
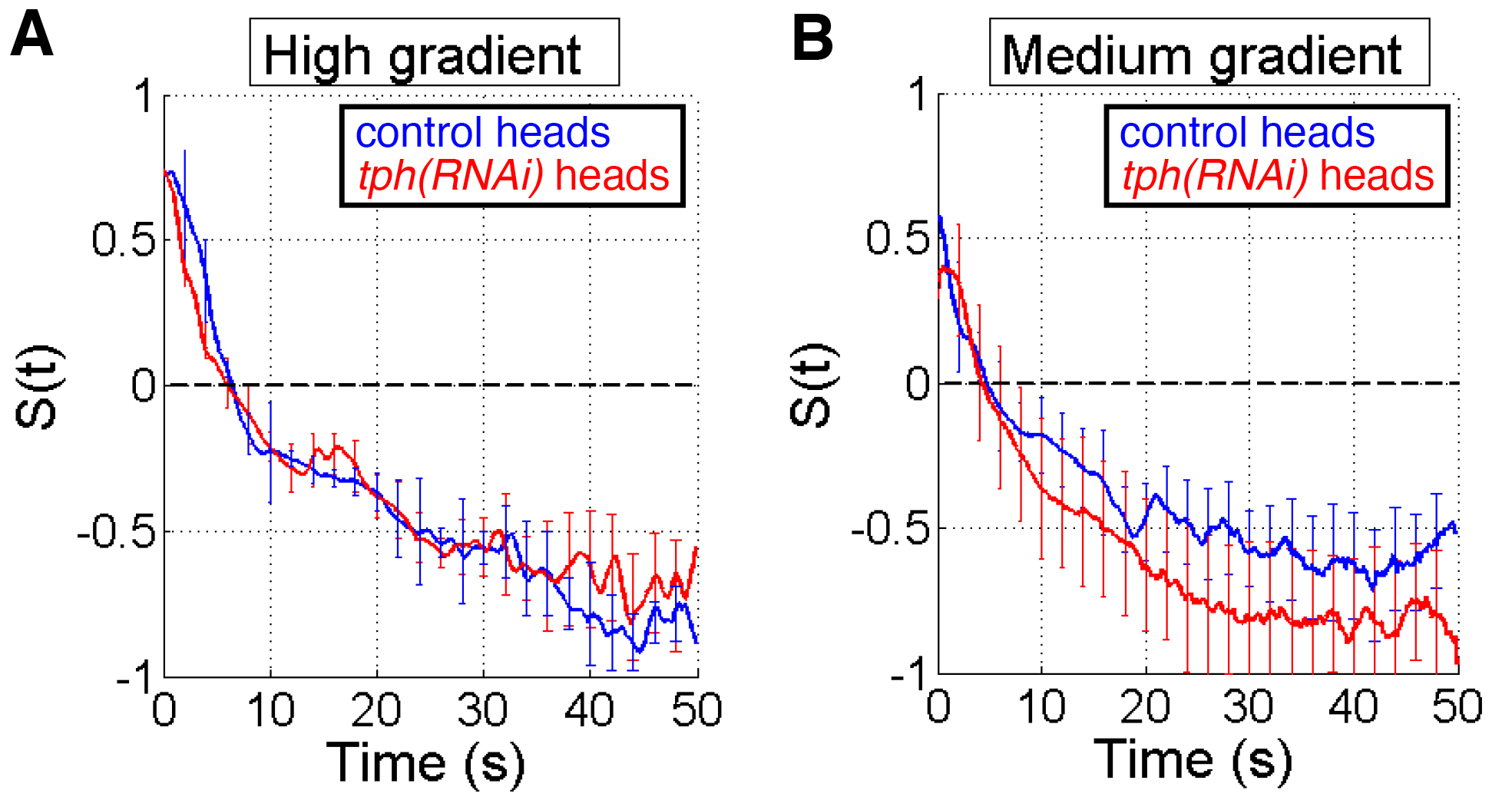
**

**S1 Fig. *tph(RNAi)* animals that retain original eye pigment do not demonstrate delayed phototaxis.** Comparison of orientation dynamics of control (blue) and *tph(RNAi)* (red) head regenerates in high (A) and medium (B) gradient settings (see Methods). *tph(RNAi)* head regenerates possess pigment cups and show similar dynamics to control worms under the two gradient settings. In contrast, the *tph(RNAi)* tails, which lack pigment cups, exhibited slower re-orientation as shown in Figure 4 in the main text. Error bars show SEM. The line at y = 0 serves as a guide to show when the worms orient away from the gradient (y < 0).
