# Supplementary material for: Tryptophan hydroxylase Is Required for Eye Melanogenesis in the Planarian Schmidtea mediterranea
Source: PLoS One. 2015 May 27;10(5):e0127074. doi: 10.1371/journal.pone.0127074 (PMC4446096; doi:10.1371/journal.pone.0127074)
Supplement: S1 Table — Measurements were taken at the front end of the arena, at the planarian start location, and at the back end. (DOCX) [file pone.0127074.s002.docx]

| **Gradient** | **Front (uW)** | **Start (uW)** | **Back (uW)** |
| --- | --- | --- | --- |
| **Low** | 10.0 ± 0.5 | 9.0 ± 0.5 | 8.0 ± 0.5 |
| **Medium** | 14.0 ± 0.5 | 11.0 ± 0.5 | 10.0 ± 0.5 |
| **High** | 23.0 ± 0.5 | 16.0 ± 0.5 | 12.0 ± 0.5 |

**S1 Table. Light intensities of gradients used in light-avoidance assay.** Measurements were taken at the front end of the arena, at the planarian start location, and at the back end.
