# Supplementary material for: Tryptophan hydroxylase Is Required for Eye Melanogenesis in the Planarian Schmidtea mediterranea
Source: PLoS One. 2015 May 27;10(5):e0127074. doi: 10.1371/journal.pone.0127074 (PMC4446096; doi:10.1371/journal.pone.0127074)
Supplement: S2 Table — A total of 10 worms per sample were run in each gradient condition. Due to boundary effects, some trajectories could not be included in the velocity analysis at the later time points. (DOCX) [file pone.0127074.s003.docx]

| **Gradient** | **ctrl RNAi** | | | ***tph* RNAi** | | |
| --- | --- | --- | --- | --- | --- | --- |
|  | **t1** | **t2** | **t3** | **t1** | **t2** | **t3** |
| **Low** | 10 | 10 | 5 | 10 | 9 | 5 |
| **Medium** | 10 | 10 | 7 | 10 | 9 | 6 |
| **High** | 10 | 10 | 8 | 10 | 10 | 9 |

**S2 Table.** **Number of trajectories used for the angular distributions at time points t1, t2, and t3 in Figure 4.** A total of 10 worms per sample were run in each gradient condition. Due to boundary effects, some trajectories could not be included in the velocity analysis at the later time points.
